# COVID-19 vaccine acceptance: knowledge and beliefs

**DOI:** 10.1186/s42269-022-00949-z

**Published:** 2022-10-24

**Authors:** Abdullah Alkattan, Nashwa Radwan, Nagla Mahmoud, Ahmed Alkhalifah, Ammar Alshamlan, Abdullah Alkamis, Amal Alfaifi, Wedad Alanazi, Amjad Alfaleh, Alhan Haji, Khaled Alabdulkareem

**Affiliations:** 1 Research Department, General Directorate of School Health, Ministry of Health, Riyadh, Saudi Arabia; 2 Department of Public Health and Community Medicine, Faculty of Medicine, Tanta University, Tanta, Egypt; 3 Department of Research, Assisting Deputyship for Primary Health Care, Ministry of Health, Riyadh, Saudi Arabia; 4 Department of Sales, Fresenius Kabi, Alhaya Medical Company, Riyadh, Saudi Arabia; 5 Department of Pharmacy, Specialized Comprehensive Polyclinics for the Security Forces, Alahsa, Saudi Arabia; 6 In-Patient Pharmacy Care Department, Prince Sultan Hospital, Melija, Saudi Arabia; 7 Department of Family Medicine, College of Medicine, Al-Imam Mohammad Bin Saud Islamic University, Riyadh, Saudi Arabia

**Keywords:** Vaccine, COVID-19, Knowledge, Beliefs, Acceptability

## Abstract

**Backgrounds:**

Vaccine acceptance varies across countries, generations, and the perceived personality of individuals. Investigating the knowledge, beliefs, and acceptability of COVID-19 vaccines among individuals is vital to ensuring adequate health system capacity and procedures and promoting the uptake of the vaccines.

**Results:**

A cross-sectional study was conducted from August 2021 to January 2022 in Saudi Arabia. The study included 281 residents to estimate their acceptance to receive COVID-19 vaccination. Around 70% of the included participants had a moderate to high COVID-19 vaccine acceptance rate during the data collection period. The risk increases to about two folds among undergraduates [OR 1.846 (1.034–3.296), *p* value = 0.036)] and increases to four folds among non-employed [OR 3.944 (2.310–6.737), *p* value = 0.001]. About 78% of participants with high and 44% with low COVID-19 vaccine acceptance (*p* value = 0.001) believed the vaccines were safe and effective. The belief that COVID-19 disease will be controlled within two years increased the risk for low vaccine acceptance by about two folds [OR 1.730 (1.035–2.891), *p* value = 0.035]. Good knowledge about COVID-19 vaccination significantly affected the acceptance rate (*p* value = 0.001).

**Conclusions:**

Several factors affect the intention of individuals to receive vaccines. Therefore, building good knowledge and health literacy through educational intervention programs, especially vaccine safety and effectiveness, is important for successful vaccination campaigns among the general population and ensuring control of the COVID-19 pandemic.

## Background

Vaccine acceptance varies across countries, generations, and the perceived personality of individuals (Habersaat and Jackson 2020; Wi Nguyen et al. 2011; La et al. 2931). Numerous studies have reported on the various aspects of new vaccine acceptance, including demographics and geographical disparities (Larson et al. 2018; Xiao and Wong 2020; Gidengil et al. 2012). A multinational survey documented by Lazarus et al. demonstrated that new vaccine acceptance in more than 18 nations varied from almost 90% to < 55% (Setbon and Raude 2010; Reiter et al. 2020; Harapan et al. 2020; Lazarus et al. 2021). The acceptance rate was 64% in the United States (US) (Horney et al. 2010), 56% in the United Kingdom (UK) (Rubin et al. 2010), 60% in Hong Kong (Chan et al. 2015), and 60% in China (Wu et al. 2018).

Concerns about vaccination need to be thoroughly understood, namely the safety and usefulness of the vaccine, knowledge, misconceptions, and confidence in the health system. Knowledge is essential to understanding pandemic hazards. Misinformation could lead to the general decline of uptake of new vaccines and jeopardize health authorities' response to the existing situation (Halpin and Reid 2022).

According to experts, at least 55% of the population should be vaccinated to achieve herd immunity against COVID-19 (Loomba et al. 2021).

Effective COVID-19 vaccination community interventions could improve the public's understanding and confidence in the vaccines and enhance their acceptance (Setbon and Raude 2010). Popular news media content should be reviewed for the most delinquent facts about COVID-19 vaccination.

Despite the ability of television and social media to rapidly revamp the community's information, nevertheless, the general awareness of COVID-19 vaccines remains low (Wang et al. 2020a).

Therefore, exploring the knowledge, beliefs, and acceptability of COVID-19 vaccines among individuals is vital to guarantee appropriate health system capacity and procedures, improving society's trust towards vaccination.

## Methods

### Study design and study participants

A cross-sectional study was conducted from August 2021 to January 2022 in Saudi Arabia. The sample size was calculated using the Cochran formula: (n = Z2 P (1- P)/d2). Where (Z) is the Standard normal variable od 1.96 at a 95% confidence interval, (n) is the minimum sample size required, P is the hypothesized proportion of acceptability of COVID-19 vaccine among the population, considered 40%, and d = acceptable margin of error, considered as 0.05. Accordingly, the estimated a minimum sample size was 384 subjects. However, it was challenging to collect the required sample, so we increased the margin of error to 0.06, and the required sample size was reduced to 256. The phone numbers of the included subjects were obtained from the 937 Medical Call Center (telemedicine service) using a systematic random sampling technique. Study participants had the following inclusion criteria: Saudi/non-Saudi individuals, males /females, vaccinated/non-vaccinated, aged 18 years and above, and responded to the questionnaire during the study period.

### Data collection

The researchers designed a new Vaccine Acceptability Questionnaire (VAQ) which experts reviewed for the validity and reliability of all its items. The basis of designing the VAQ was to develop straightforward, precise questions that focused on COVID-19 vaccines compared to some previously published surveys used to assess the COVID-19 vaccines' acceptability (Loomba et al. 2021; Setbon and Raude 2010).

The VAQ consists of 31 questions under three items: underlying factors, knowledge, and beliefs that determine the acceptability of COVID-19 vaccines. The VAQ was based on a 14-point score that ranged from 0 to 14. A score of 11–14 points was = high acceptance, 8–10.9 points = moderate acceptance, 5–7.9 = low acceptance, and less than 5 points = very low acceptability. The questionnaire was shared with the study participants through Short Message Service (SMS).

### Ethical consideration

Approval for the study was given by MOH, Institutional Review Board (IRB), Saudi Arabia (IRB log No. 21–78M). The electronic questionnaire included a note stating that responding to the questionnaire was considered an acceptance to participate in the study. The data will not be used for purposes other than the study.

### Statistical analysis

Descriptive analysis was performed using SPSS package version 24 with a level of significant difference at a *p* value < 0.05. The frequency of vaccine acceptability among studied subjects was determined. Factors associated with vaccine acceptability were studied using appropriate tests.

## Results

The study included 281 Saudi residents to evaluate their acceptance to receive COVID-19 vaccination. Their mean age was 34.4 ± 9.7 years, and 47% were female. The majority of the included study participants were undergraduates (68%), employed (60%), and had received at least one dose of any COVID-19 vaccines (95%).

Table 1 explains the acceptability rate among studied residents relative to their background variables. Around 70% of the included participants had high COVID-19 vaccine acceptance. Most participants with lower vaccine acceptance were females (56.5%), undergraduates (76.5%), and non-employed (60.0%). Female gender was a risk for low vaccine acceptance by about 1.5 folds [OR 1.766 (1.056–2.952), *p* value = 0.029]. The risk was about two folds among undergraduates [OR 1.846 (1.034–3.296), *p* value = 0.036)] and four folds among non-employed participants [OR 3.944 (2.310–6.737), *p* value = 0.001].

**Table 1:** The acceptance rate among studied participants relative to their background variables

Variable	Total participants (N = 281)	Participants with moderately-high acceptability (N = 196)	Participants with low-very low acceptability (N = 85)	*p* value [OR (CI)]
Age in years (mean ± SD)	34.4 ± 9.7	35.1 ± 10.0	32.9 ± 8.8	0.060*
Female gender (%)	131 (46.6)	83 (42.3)	48 (56.5)	0.029 [OR 1.766 (1.056–2.952)]
*Level of education*				
Less than high school (%)	15 (5.3)	13 (6.6)	2 (2.4)	0.142**
High school (%)	42( 14.9)	(34) 17.4	8 (9.4)	0.086
Undergraduate (%)	190 (67.6)	125 (63.8)	65 (76.5)	0.036 [OR 1.846 (1.034–3.296)]
Postgraduate (%)	34 (12.1)	24 (12.2)	10 (11.8)	0.909
*Job-status*				
Non-employed (%)	105 (37.4)	54 (27.6)	51 (60.0)	0.001 [OR = 3.944,(2.310–6.737)]
Employed (%)	168 (59.8)	135 (68.9)	33 (38.8)	0.001
Self-employed (%)	8 (2.8)	7 (3.6)	1 (1.2)	0.267^***^

^*^Welch’s T-test, **Chi-square test, ***Fisher exact test

Almost all residents with high vaccine acceptance (99.5%) received at least one dose of the COVID-19 vaccine, and only 9.2% had previous COVID-19 infection following vaccination. The SARS-CoV-2 infection after vaccination increases the risk of low vaccine acceptance by more than 2.5 folds compared to non-infected people [OR 2.657 (1.304–5.410, *p* value = 0.007] (see Table 2).

**Table 2:** The acceptance rate among studied participants relative to underlying factors

Underlying factors	Total N = 281	High-moderate Acceptability N = 196	Low –very low acceptability N = 85	z statistic/*p* value	OR (CI)
Got any dose of COVID-19 vaccine (%)	268 (95.4)	195 (99.5)	73 (85.9)	3.303/0.001	0.031 (0.004–0.244)
Infected with SARS-CoV-2 after getting COVID-19 vaccine (%)	36 (12.8)	18 (9.2)	18 (24.7)	2.693/0.007	2.657 (1.304–5.410)
Having a relative or a colleague infected or died because of COVID-19 (%)	154 (54.8)	109 (55.6)	45 (52.9)	0.413/0.679	1.113 (0.668–1.855)
Not given any advice from a healthcare provider to take COVID-19 vaccine (%)	153 (54.4)	103 (52.6)	50 (58.8)	0.969/0.332	0.775 (0.463–1.297)

The participants' perception of safety and efficacy significantly affected vaccine acceptance. The group with high acceptance of the COVID-19 vaccine believed it was safe and more effective than the group with low acceptance (78% vs. 44%, *p* value < 0.001).

Conversely, the belief that COVID-19 disease will be controlled within two years or more increased the risk for low vaccine acceptance among participants by about two folds [OR + 1.730 (1.035–2.891), *p* value = 0.035]. Good knowledge of COVID-19 vaccination significantly affected the acceptance rate (*p* value = 0.001) (see Table 3).

**Table 3:** The acceptance rate among studied participants relative to their knowledge and beliefs

	Total N = 281	High-moderate acceptability N = 196	Low-very low acceptability N = 85	*p* value
*Participants’ knowledge and believes*				
Participants who think that COVID-19 vaccines are mandatory	199 (70.8)	139 (70.9)	60 (70.6)	0.955
Participants who believe that COVID-19 will be controlled and finished after 2 years or more	132 (46.9)	84 (42.9)	48 (56.5)T	0.035
Participants who believe that COVID-19 vaccines are safe and effective	189 (67.3)	152 (77.6)	37 (43.5)	0.001
*Participants’ beliefs about vaccine eligibility groups*				
Children and adolescents (5–18 years)	82 (29.4)	70 (35.7)	12 (14.1)	0.001
Adults (> 18 years)	223 (79.4)	176 (89.8)	47 (55.3)	0.001
Pregnant and lactating women	121 (43.1)	109 (55.6)	12 (14.1)	0.001
Immunocompromised patients	168 (59.8)	127 (64.8)	41 (48.2)	0.009
Recovered COVID-19 patients	143 (50.9)	124 (63.3)	19 (22.4)	0.001
People diagnosed with chronic diseases	196 (69.8)	155 (79.1)	41 (48.2)	0.001
People reported any type of allergy caused by food	79 (28.1)	67 (34.2)	12 (14.1)	0.001

Table 4 reveals the acceptability rate of COVID-19 vaccination among studied residents relative to their source of knowledge.

**Table 4:** The acceptance rate among studied participants relative to different sources of knowledge

Source of knowledge	Total	High-moderate acceptability	Low-very low acceptability	*p* value
TV and radio	92 (32.7)	64 (32.7)	28 (32.9)	0.962
SMOH news	176 (62.6)	130 (66.3)	46 (54.1)	0.051
Family and friends	82 (29.2)	62 (31.6)	20 (23.5)	0.169
Social media	142 (50.5)	96 (48.9)	46 (54.1)	0.428
Healthcare providers	160 (56.9)	123 (62.8)	37 (43.5)	0.002

The group with high acceptance of the COVID-19 vaccine used official sources for vaccine knowledge, including Saudi Ministry of Health (SMOH) news and health care providers' advice than the group with low acceptance (66% vs. 63% and 54% vs. 44%, respectively).

## Discussion

It is essential to ensure the availability and efficacy of vaccines to control the COVID-19 pandemic successfully. Meanwhile, health authorities should ensure community trust and acceptance of the vaccine. Any hesitation and vaccination refusal could hinder the healthcare system's ability to control the pandemic.

The current study investigated factors associated with vaccine acceptance among studied residents. The reported background variables associated with a low acceptability rate are low education, non-employment, and female gender. This finding was inconsistent with Nehal et al., who found that gender and education were not significant variables for vaccine acceptance (Nehal et al. 2021).

The present study showed that about 70% of the participants reported a moderate to high acceptance of the COVID-19 vaccine. Several other Saudi studies were almost in line with our results, which showed that 62–71% of Saudi citizens and Saudi residents have a good acceptance of COVID-19 vaccines (Narapureddy et al. 2021; Alqahtani 2021; Alshahrani et al. 2021; Maqsood et al. 2022; Al-Mohaithef and Padhi 2020; Fadhel 2021; Yahia et al. 2021; Elharake et al. 2021). However, Khalafalla et al. reported that 83.6% of Jazan University students were willing to take the COVID-19 vaccine, which was described as having a high acceptance rate compared to most studies conducted in Saudi Arabia (Khalafalla et al. 2022). Despite that, the results revealed by Khalafalla et al. could not reflect the general population as they included a very specific group of people.

Nevertheless, a systematic review of 33 countries found that vaccine acceptance varies between countries with different socio-economic status. Low acceptance was reported in countries such as Kuwait (23.6%) and Jordan (28.4%), and moderate acceptance in countries such as Italy (53.7%), Poland (56.3%), and Russia (54.9%). In contrast, some countries exhibited high acceptance, especially in East Asia, such as Indonesia (93.3%), China (91.3%), and Malaysia (94.3%) (Sallam 2021).

Despite the misinformation and rumors about COVID-19 vaccines repeatedly shared on social media platforms (Puri et al. 2020), about half (51%) of the studied participants depended on social media as their primary source of knowledge. A study reported that vaccine hesitancy and refusal are associated with misinformation on social media (Bianco et al. 2019). This finding reflects the importance of educational interventions to address misinformation about vaccines.

Likewise, participants' perception of the vaccine as safe and effective was another significant factor in the high acceptance rate among studied participants. Conversely, the belief that COVID-19 disease will be controlled within two years and COVID-19 infection after vaccination were inversely associated with the vaccine acceptance rate. Similarly, a study in China found that 48% of respondents postponed vaccination because they were concerned about the safety of vaccines (Wang et al. 2020b). Also, a United States study found that 89% of participants assumed that COVID-19 vaccines could have some side effects which affected vaccine acceptance (Callaghan et al. 2020).

While a survey in Bangladesh found that about a quarter of participants thought that COVID-19 was safe, only 60% were willing to be vaccinated, and about two-thirds will recommend it to family and friends (Islam et al. 2021). It is believed that misinformation and lack of data on the seriousness of incidence and mortality of COVID-19 disease may reduce concerns about vaccine acceptability (Geoghegan et al. 2020).

Inconsistent with the current study findings, Elhadi et al. found that having a family member or friend infected with COVID-19 was positively associated with the likelihood of vaccine acceptance (OR 1.09 [1.02, 1.18]). While they found that having a friend or family member who died due to COVID-19 was negatively associated with it (OR 0.89 [0.84, 0.97]) (Elhadi et al. 2021).

Likewise, a meta-regression analysis conducted by Kukreti et al. found that a COVID-19 death rate of 400 per million population or higher was significantly associated (*p* value = 0.02) with the willingness rate for vaccination (Kukreti et al. 2022).

The variation in the acceptability rate between different studies reflects the interaction between vaccine perception and background variables, namely age groups and educational levels.

## Conclusions

Several factors affect the intention of individuals to receive vaccines. Therefore, building good knowledge and health literacy through educational intervention programs, especially vaccine safety and effectiveness, is important for successful vaccination campaigns among the general population and ensuring control of the COVID-19 pandemic.

## Data Availability

The datasets used and/or analyzed during the current study available from the corresponding author on reasonable request.

## Abbreviations

CI: Confidence interval
COVID-19: Coronavirus disease 2019
IRB: Institutional Review Board
MOH: Ministry of Health
OR: Odds ratio
SARS-CoV-2: Severe acute respiratory syndrome coronavirus 2
SD: Standard deviation
SMOH: Saudi Ministry of Health
SMS: Short Message Service
TV: Television
US: United States
UK: United Kingdom
VAQ: Vaccine Acceptability Questionnaire
